# Scabies: Immunopathogenesis and pathological changes

**DOI:** 10.1007/s00436-024-08173-6

**Published:** 2024-03-04

**Authors:** Mahmoud S. Sharaf

**Affiliations:** Parasitology Department, Faculty of Medicine, Tanta University, Tanta, Gharbia Egypt

**Keywords:** Scabies, *Sacrcoptes scabiei*, Immunopathogenesis, Pathology, Systemic changes

## Abstract

Scabies is an itchy skin disease caused by the burrowing mite *Sarcoptes scabiei*. During their lifespan, the female mites invade the stratum corneum and create tunnels, in which they reside, move, feed, deposit fecal pellets, and lay eggs. Recently, scabies was included in the World Health Organization roadmap for neglected tropical diseases 2021–2030. This review attempts to summarize our knowledge about the mite’s biology and the disease pathogenesis, pathological changes, and complications. Generally, the host–parasite interaction in scabies is highly complex and involves different mechanisms, some of which are yet largely unknown. Elucidation of the nature of such interaction as well as the underlying mechanisms could allow a better understanding of the mite’s biology and the development of novel diagnostic and therapeutic options for scabies control programs. Moreover, identification of the molecular basis of such interaction could unveil novel targets for acaricidal agents and vaccines.

## Background

Scabies is a skin disease caused by a microscopic burrowing mite called *Sarcoptes scabiei* (*S. scabiei*). Recently, it was included in the world health organization (WHO) roadmap for the neglected tropical diseases (NTDs) 2021–2030, since it fulfills the recently specified WHO inclusion criteria for NTDs, being disproportionately influencing poor population, causing significant morbidity and mortality, mainly affecting people in tropics and subtropics, relatively neglected by researchers, and liable for control, elimination, or eradication (WHO 2020).

Regardless of the frequent reports indicating high prevalence, scabies is rarely prioritized in health control programs and research, possibly because disease complications span a wide range of disciplines such as parasitology, dermatology, immunology, infectious diseases, veterinary science, and disease ecology (Takano et al. 2023).

## Epidemiology

Unfortunately, there are no available accurate current estimates regarding the global prevalence of scabies owing to the paucity of epidemiological data (Engelman et al. 2020). In the 2015 Global Burden of Disease Study, the global prevalence of scabies was estimated to affect more than 200 million individuals (Karimkhani et al. 2015). While scabies could affect people of all ages globally, children and the elderly in low-resource areas are the most vulnerable. The tropical countries have the highest prevalence for scabies. However, scabies is not confined to the tropical countries. Cold weather may be associated with increased incidence of scabies, which can be explained by increased direct personal contact and increased mites’ survival (Liu et al. 2016; El-Moamly 2021). Equally important, prolonged direct contact and overcrowding increase the risk of transmission and endemicity of scabies. So, scabies is an indirect reflection of poverty and overcrowding, rather than a disease of poor hygiene. This explains the high incidence of scabies in prisons, military camps, and boarding schools (Heukelbach et al. 2005; Alsamarai 2009).

Although both developing and developed countries are affected, scabies tends to be endemic in the resource-poor countries, with a higher morbidity and mortality. Sometimes, it can affect 20% or more of the whole population (Hay et al. 2012; Heukelbach et al. 2013). On the contrary, in the high-income countries, it usually occurs sporadically or in outbreaks in localities where people live in close quarters such as hospitals, nursing homes, prisons, childcare facilities and institutions for elderly (Ariza et al. 2012; Makigami et al. 2012).

## Biology of *Sarcoptes scabiei* mite

### Taxonomy and variants

Previously, *Sarcoptes scabiei* was known as *Acarus scabiei*, which was classified as a member of the genus *Acarus*. Later, it was placed in the genus *Sarcoptes*, which belongs to the superfamily Sarcoptoidea and the family Sarcoptidae (Arlian and Morgan 2017). *Sarcoptes scabiei* can be classified according to Schoch et al. (2020) as shown in (Table 1). Several variants of *S. scabiei* mites have been reported to infect humans and a wide range of mammals (e.g., *S. scabiei* var. *canis* for dogs, *S. scabiei* var. *cuniculi* for rabbits, *S. scabiei* var. *vulpes* for red fox and *S. scabiei* var. *hominis* for humans). Most authors suggest that genus *Sarcoptes* includes only one valid species with different varieties exhibiting different host-specific preferences (Arlian and Morgan 2017).

**Table 1:** Taxonomy of *Sarcoptes scabiei* (Schoch et al. 2020)

Superkingdom:	Eukaryota
Kingdom:	Metazoa
Phylum:	Arthropoda
Subphylum:	Chelicerata
Class:	Arachnida
Subclass:	Acari
Superorder:	Acariformes
Order:	Sarcoptiformes
Suborder:	Astigmata
Parvorder:	Psoroptidia
Superfamily:	Sarcoptoidea
Family:	Sarcoptidae
Subfamily:	Sarcoptinae
Genus:	*Sarcoptes*
Species:	*Sarcoptes scabiei*

### Morphology (Fig. 1)

**Fig. 1:**
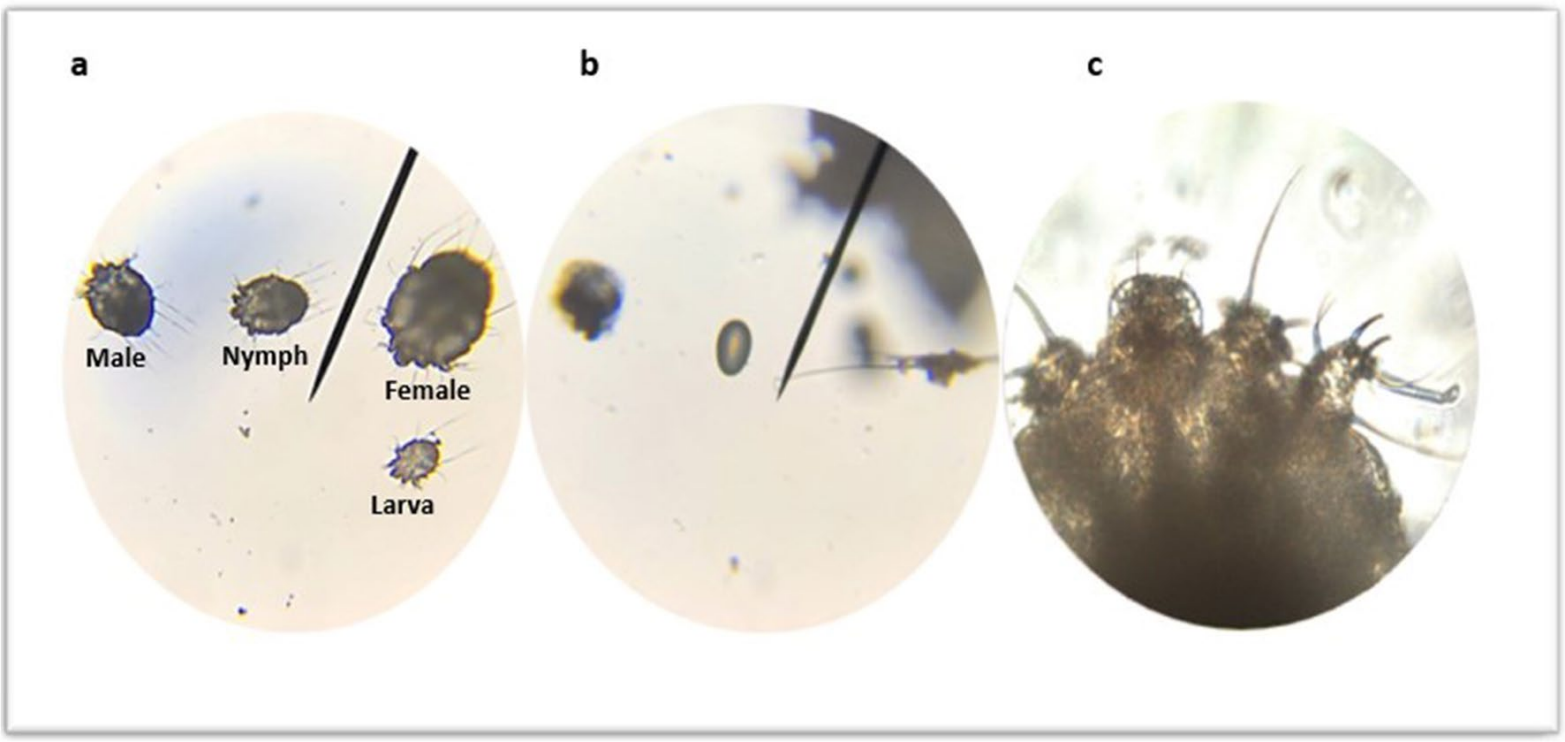
Microscopic appearance of *S. scabiei*. Figure (a) shows all motile stages of the mite (× 100), figure (b) shows egg of the mite (× 100), while figure (c) shows a magnified anterior end of the female mite dorsal surface (× 400)

*Sarcoptes scabiei* mite has a characteristic tortoise-like body. Its body shows the gnathosoma anteriorly, and the idiosoma posteriorly. The gnathosoma is a head-like structure, including the chelicerae and the pedipalps, that surround the mouth opening. The idiosoma is oval, ventrally flat, and dorsally arched, with four pairs of legs arising from the ventral aspect of the adult mites (Arlian and Morgan 2017). The dorsal surface of the idiosoma is not smooth. It shows transversely arranged ridges, scales, setae, and spines.

The female measures approximately 0.30–0.45 mm in length and 0.25–0.35 mm in width, while the male measures approximately 0.21–0.28 mm in length and 0.16–0.21 mm in width (Banzhaf et al. 2013). The eight legs of the adult mite are arranged in two sets, each set of them includes two pairs of legs which have similar morphology. Instead of the terminal bristle on the fourth leg, the male bears a sucker. This feature distinguishes males from females, and it appears to help the male mite during copulation in grabbing the female mite as these suckers are found near the genitalia (Klompen 1992).

*Sarcoptes scabiei* eggs are ovoid and measure about 0.10 to 0.15 mm in length. Identifying the unique morphological and biological features of the egg could help the researchers to understand why most available medications for scabies have little effect on this stage. The protective eggshell could be the first barrier that prevents current scabicides from reaching its target (Bernigaud et al. 2019).

The larva of the *S. scabiei* mite is the smallest ambulatory stage of the mite (about 200 µm long and 155 µm wide) that immediately hatches from the egg. It has only six legs. The nymph is the largest immature stage just prior to the final molt. It is smaller than the adult female and about the same size as the adult male (Arlian and Morgan 2017).

### Life cycle

*Sarcoptes scabiei* has five developmental stages in its life cycle: egg, larva, protonymph, tritonymph, and adult. Adults mate on the skin of the host, and males search the skin for the unfertilized females for several days after mating. During their lifespan, which is about 4–6 weeks, the females lay 2–4 eggs per day in a burrow in the stratum corneum. Two to four days after the egg has been laid, a larva with sex legs emerges. The larva digs a new pocket burrow to leave the burrow. It wanders on the surface of the host's body for 14–17 days till maturation. Maturation occurs as the larva moults into the eight-legged protonymph, tritonymph, and then the adult male or female (Kraabol et al. 2015).

### Survival, infectivity, and transmission between different hosts

Prolonged direct contact, including sexual contact, with a host infected by *S. scabiei* mite is generally considered to be the main mode of transmission of scabies. However, the host-seeking behavior of the mite could allow indirect transmission via fomites like bed linens, towels, and clothes also (Walton and Currie 2007).

The indirect transmission is largely affected by the ability of the mite to survive and to remain infective while off the host in the external environment, which in turn is affected by the environmental temperature and relative humidity. Higher temperatures (even at high humidity) are associated with shorter survival time of the mite. In these conditions, *S. scabiei* mite usually dies due to its inability to maintain water balance in its body. In contrast, higher relative humidities and lower temperatures allow longer survival time for the mite. Generally, all life stages could survive for one to nine days at 15–25° C and 25- 97% relative humidity (Arlian and Morgan 2017).

Regarding infectivity, experiments show that scabies mite could keep its infectivity for at least one half to two thirds of its survival time in the external environment off its host. For example, at room conditions, *S. scabiei* var. *suis* was found to remain infective for about 24 h after death of its host (pig). Along similar lines, *S. scabiei* var*. canis* retain its ability to infect new hosts for about 36 h after death of its host (Alasaad et al. 2013).

### Skin penetration, feeding and water balance maintenance

When *S. scabiei* infects a host, it starts secreting saliva that forms a pool around their body to lyse the stratum corneum of the epidermis. As the mite sinks into the stratum corneum, the anterior two pairs of legs move in a tortoise-like pattern (i.e., swimming motion) to advance forward within the tunnel. The mite imbibes its water needs from the host intercellular fluid (lymph) that leaks into the burrow around the mite mouthparts when they burrow deep in the stratum corneum near the lower living epidermal layers (Arlian and Morgan 2017). Detection of the host’s IgG antibody in freshly separated mites’ midgut is clear evidence that mites consume the serum of the host (Rapp et al. 2006).

## Host immune response to *Sarcoptes scabiei*

Within the epidermis, the first functional immune cells to be stimulated by the scabies mite and its products are keratinocytes, dendritic cells, and Langerhans cells (LCs) (Bhat et al. 2017). When the mite’s products reach the dermis, they stimulate fibroblasts, microvasculature endothelial cells, and immunological effector cells such as LCs, macrophages, mast cells, and lymphocytes. The antigens from the mites in the skin are likely taken up and processed by the LCs and other dendritic cells, which then transport them to the regional lymphatic tissue, where B- and T-lymphocytes are activated, triggering an adaptive immune response (Walton 2010).

### Innate immune response

#### Innate immune cells

Dermal infiltration with eosinophils is an integral part of the host immune response to *S. scabiei,* especially in CS, possibly due to the high expression of interleukin-5 (IL-5). The recruitment, activation, and maturation of eosinophils are all aided by IL-5. Even peripheral eosinophilia has been reported in CS (Roberts et al. 2005; Jayaraj et al. 2011; Bhat et al. 2017).

The exact role of eosinophils in ordinary scabies (OS) and CS is still unknown. They may control the T helper-2 (Th2) response through expressing some Th2 cytokines (Walton et al. 2010). Also, they may influence the Th1 response through synthesis of IL-12 and interferon gamma (IFN-γ), as well as expression of many toll-like receptors (TLRs) as TLR-7 (Nagase et al. 2003). Furthermore, the eosinophil expression of IL-10 and transforming growth factor beta (TGF-β) may reduce the local inflammatory responses by influencing the activity and growth of regulatory T cells (Jacobsen et al. 2007).

The dermal infiltration with mast cells and basophils has been reported in scabies, especially in CS. Upon activation, they produce tumor necrosis factor alpha (TNF-α), IL-6, and Th2 cytokines, including IL-4, IL-5 and IL-13 (i.e., main cytokines in allergic Th2 response) (Prussin and Metcalfe 2006; Schroeder 2011).

Macrophages, neutrophils and dendritic cells are immune effector cells involved in phagocytosis, antigen presentation, differentiation of T-cells, and proinflammatory responses. Various degrees of dermal infiltration by these cells have been reported in scabies. Their exact roles in the immune and inflammatory responses in scabies need further investigations (Bhat et al. 2017).

#### Complement system

The detection of C3a and C4a in skin biopsies from affected hosts may imply that complement system is involved in the immune response to the scabies mites (Roberts et al. 2005; Walton et al. 2008). Surprisingly, low serum C3, C4, or both have been reported in CS, suggesting a potential defect in the complement function, or system failure due to massive overload of mites and bacteria. Both C3a and C5a can activate mast cells, leading to release of the inflammatory mediators such as histamine and TNF-α (Roberts et al. 2005).

### Adaptive immune responses

According to the type and magnitude of the elicited immune response, two major forms of scabies are recognized: OS and crusted scabies (CS) (Walton and Oprescu 2013). Ordinary scabies has been proved to be associated with a protective local immune response that is dominated by cluster of differentiation (CD) 4 + T-lymphocytes with a Th1 cytokine profile (i.e., IFN-γ, TNF-α and IL-2) (Liu et al. 2014). In contrast, the immune response in CS has been proved to be dominated by CD8 + T-lymphocytes, with a non-protective Th2 cytokine profile (i.e., IL-4, IL-5 and IL-13) (Walton et al. 2008; Liu et al. 2014). Th17 cells are another major subset of T-cells that share in the local immune response in cases of CS by recruiting and activating neutrophils at sites of inflammation. Also, they stimulate endothelial cells, keratinocytes, and fibroblasts to produce inflammatory cytokines, like IL-1, IL-6, and TNF-α. Their role in CS is evidenced by high levels of IL-17 and IL-23 (Bhat et al. 2017).

Regulatory T-lymphocytes (Treg) also may have a role in the control or development of scabies. They may inhibit the inflammatory and immune reactions to the parasite via secretion of IL-10 and inhibition of synthesis of the proinflammatory cytokines like TNF-α, IFN-γ and IL-2 (Liu et al. 2014). This may partially explain the incubation period in a primary infestation by *S. scabiei.* There is a negative correlation between activity of Treg cells and severity of lesions in scabies (Abd El-Aal et al. 2016). This explains why skin pathology is severe in CS, in which IL-10 production is significantly reduced, compared to OS, leading to expansion of Th17 cells and a Treg/Th17 dysfunctional immune response (Gonzalez-Lombana et al. 2013).

Infestation with *S. scabiei* mites could trigger strong antibody-mediated immune responses, particularly in CS, which has extremely high levels of antigen-specific IgG and IgE when compared with OS. This difference could be explained by the high expression of IL-4 and IL-13 in CS. Interleukin-4 and IL-13 play crucial roles in B cell class switching and induction of IgE and IgG4 co-expression. Unfortunately, timing of the antibody-mediated immune responses, as well as their relative importance in establishing protective immunity, are still largely unknown (Bhat et al. 2017).

## Pathogenesis of scabies (Fig. 2)

**Fig. 2:**
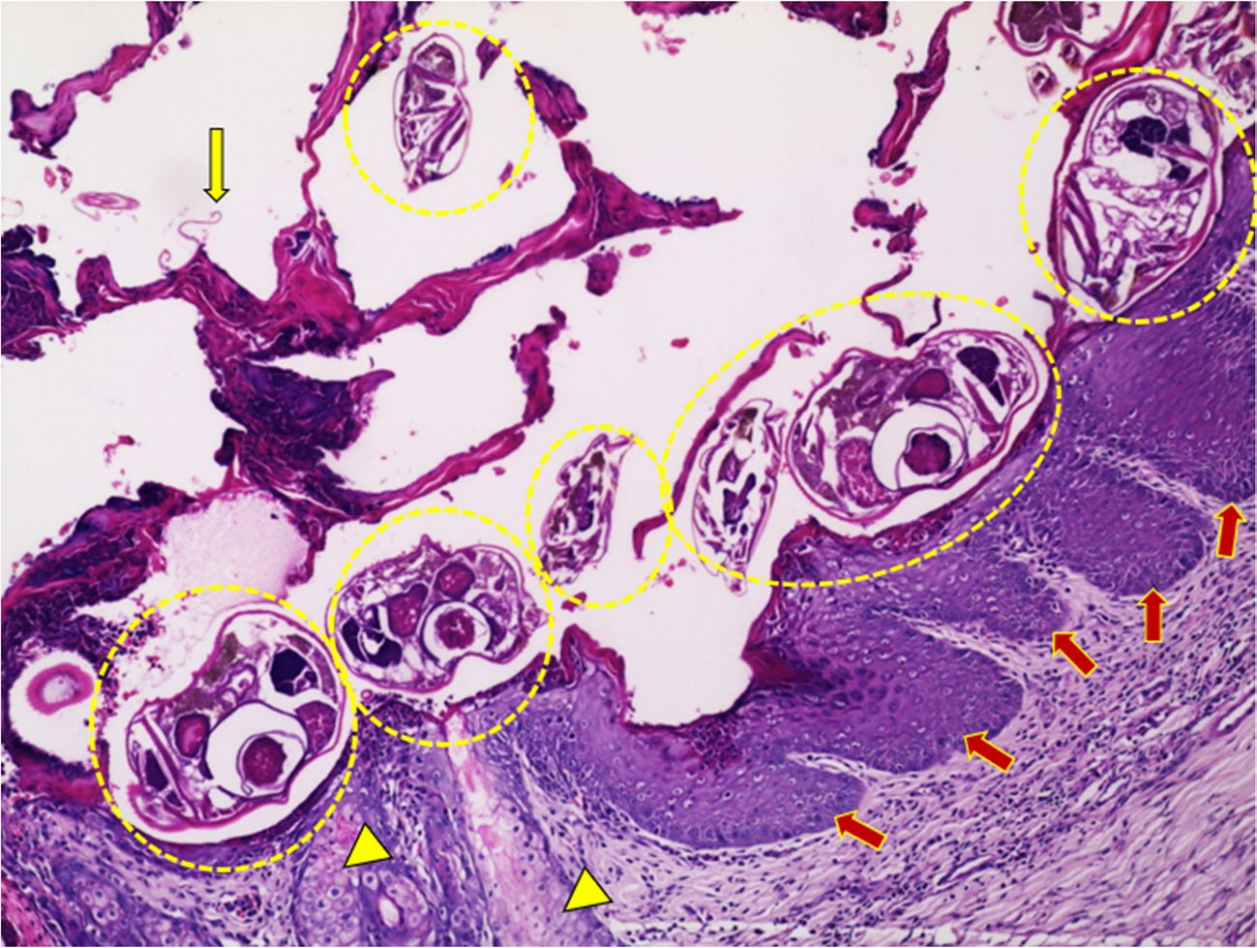
Histopathological changes in crusted scabies. Note the pigtails sign which represents the empty eggshells “yellow arrow”, heavy mite infestation “yellow dashed circles”, rete ridges hypertrophy “red arrows”, sebaceous glands hypertrophy “yellow arrowhead”, and diffuse heavy dermal inflammatory infiltration (Hematoxylin and eosin, × 100)

The host–parasite interaction in scabies is highly complex and involves different interconnected factors, some of which are yet largely unknown, and it is not easy to distinguish the contribution of each factor separately. Generally, scabies pathogenesis could be summarized as follows:

### Triggering the cascading pathogenic consequences by *S. scabiei*

The mites invade the stratum corneum of the epidermis and create tunnels, in which they reside, move, feed, deposit fecal pellets and lay eggs. Using the cutting mouthparts and hooks on their legs, the mites burrow deeply in the stratum corneum near the lower living epidermal layers. To facilitate burrowing and feeding, mites release substances that may aid in lysis of the host tissue (Bornstein et al. 2001; Pence and Ueckermann 2002). Being close enough to the lymph in their residence in the epidermis, the lymph could flow into the burrow and supply the mites with nutrients and water needs. The soluble mite antigens, released from both viable and dead mites, can then diffuse into the dermis, triggering an immunological and inflammatory response in the host (Arlian and Morgan 2017). The role of dead mites in the pathogenesis of scabies explains why pruritus persists for some time after treating scabies (Zhu et al. 2004; Elder et al. 2009).

### Immunopathogenesis

The main manifestations of scabies are mediated through the inflammatory and allergic responses mounted by the host against the mite products that are deposited under the skin. Both immediate (type I) and delayed (DTH or type IV) hypersensitivity responses are involved (Walton et al. 2008; Bhat et al. 2017). Although the exact role of cell-mediated and humoral host immune responses in the pathogenesis of OS and CS remains largely unknown, it is now well established that clinical severity of the disease depends on variations in the type and amplitude of the associated cellular and humoral responses (Gazi et al. 2022).

The presence of T lymphocytes in the locally evoked immune response against *S. scabiei* is an integral feature that generally indicates development of DTH reaction, which could be elicited by either CD4 + or CD8 + T-cells. They release mediators that stimulate local endothelial cells and enhance recruitment of inflammatory cells, causing lesion development (Yalew 2020; Doukas et al. 2021). Like atopic dermatitis, the dermal lymphocytic infiltrate in OS is dominated by CD4 + T-cells, with a reported CD4/CD8 ratio of 4:1. In contrast, CD8 + T-cells dominate the dermal lymphocytic infiltration in CS, with minimal or no CD4 + T-cells. Similar findings have been reported in psoriasis, a disease characterized by erythematous scaly papules, plaques, and abnormal keratinocyte hyperproliferation (Walton et al. 2008; Bhat et al. 2020).

Although the exact role of CD8 + T cells in CS is unspecified, they may induce tissue damage by exhibiting direct cytotoxicity against keratinocytes and/or releasing cytokines, which could enhance the inflammatory response targeting resident skin cells (Hay et al. 2012; Bhat et al. 2017; Gazi et al. 2022). Th17 cells are another major subset of T-cells that share in the pathogenesis of CS by recruiting and activating neutrophils at sites of inflammation. Also, they stimulate endothelial and epithelial cells to produce inflammatory cytokines, like IL-1, IL-6, and TNF-α (Bhat et al. 2017).

Since it has been described as one of the most prominent cytokines in CS skin lesions, interleukin-1β (IL-1β) appears to be a crucial cytokine in the pathogenesis of CS. It is involved in the differentiation and activation of T lymphocytes, including Th17. Furthermore, it has been reported to have a synergistic effect with TNF-α, thus triggering the inflammatory cascade by IL-1β and TNF-α could result in inflammation, tissue damage, and loss of function (Walton et al. 2008; Bhat et al. 2020). Interestingly, IL-1β has also been incriminated to be a key player in the pathogenesis of psoriasis (Cai et al. 2019).

Transforming growth factor-β, IL-4, IL-10 and IL-13 are among cytokines implicated in the pathogenesis of CS. They inhibit macrophage activation. Also, TGF-β could suppress the inflammatory Th1 response. Failure to evoke an effective immune response could explain the extensive numbers of mites over the body of the host. Along similar lines, IL-4 could trigger keratinocytes proliferation, since epidermal cells have IL-4 receptors. Interestingly, IL-4R expression has been reported to be upregulated in psoriasis. IL-4 could also contribute to the excessive IgE synthesis observed in CS (Walton 2010; Hashim et al. 2020).

Although *S. scabiei* can elicit a strong humoral immune response in CS, antibodies do not appear to be protective in scabies. Specific IgE, on the other hand, plays a significant role in the pathogenesis of allergic diseases (Abd El-Aal et al. 2016). Notably, IgE response is frequently accompanied by IgG4 production. The precise functions of IgG4 in scabies are still questionable, however it may protect the host against anaphylactic reactions by serving as blocking antibodies (i.e., block IgE receptor sites on antigens) (Walton et al. 2010; Scott-Taylor et al. 2018).

Despite the high serum levels of IgE, IgG, and IgG subclasses in CS, B lymphocytes are typically absent in dermal inflammatory infiltrate. This may be a contributing factor that explains the failure of the locally evoked immune response to protect the host against the mites. Furthermore, skin does not contribute to the high levels of serum immunoglobulins in CS (Doukas et al. 2021). In contrast to CS, B-cell infiltration and activation have been described in OS skin lesions, with deposition of IgM and IgA at the dermal–epidermal junction. Furthermore, C3 deposition within dermal blood vessels has been described. Hence, local inflammatory responses in OS skin lesions could be mediated through deposition of *S. scabiei*-specific antigen–antibody complexes and complement activation (Walton et al. 2008).

Scabies has traditionally been referred to as "the worst itch" a patient could ever experience, highlighting the excruciating itching that occurs in this disease. Such itching can be caused by either the scabies mite's direct action or the host immune response to it. *Sarcoptes scabiei* can trigger itching through activation of TLR-3,4 and 7 expressed on primary sensory neurons (Taves and Ji 2015). Furthermore, proteases present in mite feces could stimulate protease-activated receptor-2 (PAR-2), a known pruritic receptor, on keratinocytes (Lavery et al. 2016).

Moreover, itching may be induced by the immune response developed by the host against the mite through different possible mechanisms. The first mechanism involves complement system activation, which in turn activates mast cells, causing release of histamine, tryptase, and TNF-alpha (Ricklin et al. 2010; Misery 2014). The second mechanism involves activation of macrophages, resulting in production of prostaglandins and leukotrienes that potentiate the itch. Furthermore, the Th1 response in classic scabies is associated with production of IFN- γ, TNF- α, and IL-2 that activate pruriceptors, whereas the Th2 response in CS is associated with activation of pruriceptors via B-cell activation, production of IL-4, IL-5, IL-13, and IL-31, and IgE-mediated mast cell activation (Misery 2014; Kim et al. 2021).

### Modulation of the host inflammatory and immune responses by *S. scabiei*

Tissue-feeding parasites, like *S. scabiei*, confront significant threats that endanger their early survival, being exposed to the host’s immune mechanisms both internally (by the ingested epidermal proteins and plasma) and externally. As a result of a long co-evolution with its mammalian hosts, the mite has evolved several mechanisms to evade the host's innate and adaptive immune responses (Hay et al. 2012).

Although hosts exhibit different immune mechanisms to fight the mite, as mentioned above, clinical symptoms of a primary *S. scabiei* infestation can take up to four to eight weeks to appear (Morgan et al. 2013). Evidence suggests that the mites produce chemicals that inhibit the host's early inflammatory and immune responses. This delay is thought to give the mite population time to establish in the host before a strong defense response is elicited. Inflammatory and immune reactions, on the other hand, appear more rapidly in sensitized hosts (Elder et al. 2009).

Live scabies mites have been reported to trigger cells in human skin equivalents, like fibroblasts and keratinocytes, to secrete the anti-inflammatory cytokine interleukin-1 receptor antagonist (IL-1ra). By binding to the IL-1 receptor, which is located on numerous cells including T-cells, B-cells, natural killer cells, macrophages, and neutrophils, IL-1ra inhibits the function of the proinflammatory cytokine IL-1 (Morgan and Arlian 2010).

Along similar lines, *S. scabiei* mite extract has been reported to inhibit the production of intercellular and vascular cell adhesion molecules as well as E-selectin in cultured normal human endothelial cells of the skin microvasculature. As a result, extravasation of lymphocytes, neutrophils, and other cells into the dermis would be inhibited or diminished. Consequently, the host's capacity to evoke a successful protective response would be hampered (Elder et al. 2006).

Human T-regulatory cells have been reported to be stimulated to generate IL-10 by scabies mite extract. By decreasing the release of proinflammatory cytokines and the expression of MHC-II molecules on antigen-presenting cells, IL-10 serves as a strong anti-inflammatory cytokine. Thus, the connection between the MHC-II-antigen complex and the T-cell receptor, which is required for B-cell activation and proliferation into antibody-secreting plasma cells, would be inhibited/reduced (Arlian et al 2006).

In response to live scabies mites and mite extracts, human skin equivalents and monocultures of normal human epidermal keratinocytes and dermal fibroblasts have been reported to increase production of vascular endothelial growth factor. The vascular endothelial growth factor would enhance vascularity and fluid (plasma) entry to the mite burrow around the mite's mouthparts. This fluid is the mite's primary source of water and nutrients in the otherwise dry stratum corneum (Arlian et al. 2003; Morgan and Arlian 2010).

Interference with IL-8 activity is another mechanism by which the mite can modulate immune response of the host. Interlukin-8 is a chemokine that is chemotactic for neutrophil extravasation to the location of a pathogen (Mullins et al. 2009). When compared to unchallenged controls, monocultures of human epidermal keratinocytes, dermal fibroblasts, microvascular endothelial cells of the skin, and dendritic cells challenged with scabies extract showed lower levels of IL-8 in the medium (Arlian et al. 2003, 2004; Elder et al. 2006). Furthermore, scabies mites can produce IL-8 binding protein that reduces local IL-8 levels, hence limiting neutrophil chemotaxis (Mullins et al. 2009).

*Sarcoptes scabiei* have been reported to use gut serine protease inhibitors to bind to many plasma proteins involved in the complement activation pathways and block the three pathways of the human complement system (classical, alternative, and lectin). Since mites consume plasma, inactivating host complement may protect the mite gut from complement-mediated damage (Rapp et al. 2006). Complement suppression may enhance the pyoderma produced by group A streptococci, which is frequently linked with scabies lesions (Mika et al. 2012a, b).

### Sarcoptes scabiei and secondary bacterial infections

Hosts infected with *S. scabiei* are often particularly susceptible to 2ry bacterial infection, being undernourished and immunologically disarmed. As a result, complications like cellulitis, lymphangitis and acute glomerulonephritis could occur. Death may occur in severe cases (Kemp et al. 2002). In humans, *Staphylococcus aureus* and group A haemolytic streptococci are the most common examples reported in scabies (Brook 2002). Interestingly, they have been isolated from skin burrows as well as mite fecal pellets, suggesting that scabies mites may aid in the transmission of pathogenic bacteria within the host (McCarthy et al. 2004).

### *Sarcoptes scabiei* and oxidative stress

Free radicals form an integral part in the host's defense against the parasites, but when they are produced in large quantities, the antioxidant mechanisms become overwhelmed, and the generated free radicals can then interact with endogenous macromolecules, resulting in damage to DNA, lipids, and proteins, as well as inducing and/or enhancing tissue injury (Pizzino et al. 2017). The proinflammatory cytokines induced by *S. scabiei*, including IL-1, IL-6, IL-17, TNF-α and IFN- γ, can lead to excessive generation of free radicals. Recent findings suggest that oxidative stress-induced tissue damage plays an important role in the pathogenesis of many skin disorders. Free radicals can then induce or aggravate skin lesions in scabies such as erythema, inflammation, and abnormal keratinization (Bickers and Athar 2006). Recently, *S. scabiei* has been reported to cause oxidative stress in mammalian hosts (Abu Hafsa et al. 2021; Sharaf et al. 2023a).

## Histopathological changes in scabies

### Local changes

While there is a considerable histopathologic overlap between different clinical forms of scabies, there are some characteristics that are more distinctive to each (Elwood et al. 2015).

#### Ordinary scabies

Macroscopically, lesions usually appear within four to eight weeks after primary infestation by *S. scabiei*. However, they can appear as soon as one to two days in case of secondary infestation (McCarthy et al. 2004). The burrow is the pathognomonic lesion for scabies. It is a short, straight track that ends in an intact vesicle or erosion harboring the mite. It is usually found in the web spaces of the fingers, hands, wrists, axillae, feet, buttocks, and genitalia. Although the presence of burrows is pathognomonic for scabies, they are only seen in a few cases. Instead, nonspecific secondary lesions such as excoriated papules, eczematous or lichenified plaques, nodules are more commonly seen (Cassell et al. 2018).

Microscopically, the ordinary type is typically characterized by irregular acanthosis, spongiosis and exocytosis of eosinophils and neutrophils. Occasionally, intraepidermal microabscesses are formed. Older lesions may show parakeratosis and signs of excoriation such as serum crusts (Hicks and Elston 2009). Deeply within the stratum corneum of the epidermis, burrows containing adult females, eggs, eggshells, larvae and/or fecal deposits could be seen.

Adult female could be identified by its characteristic oval body with thin exoskeleton and dorsal spines (Mathison and Pritt 2014). Even in cases in which the entire mite is not visible, curled pink structures that have been referred to 'pigtails' are a very valuable clue in the stratum corneum. These structures are the remains of eggs that have been left behind after hatching (Cardoso et al. 2017).

Regarding dermal changes, superficial or deep perivascular and interstitial mixed inflammatory cell infiltration, including lymphocytes, histiocytes, and eosinophils, is commonly seen (Hengge et al. 2006). Although vascular changes are not common, endothelial swelling, luminal narrowing, vasculitis and fibrinoid deposition within and around vessels may occur (Elwood et al. 2015).

#### Nodular scabies

This type of scabies may develop in patients with chronic infestation, probably due to hypersensitivity. Macroscopically, it is characterized by severe eczematous changes and highly pruritic nodules, especially on male genitalia and breasts, that persist for weeks or months even after effective treatment (Sil et al. 2020). Microscopically, nodular scabies is characterized by dense perivascular inflammatory infiltrations with lymphocytes, histiocytes, eosinophils, plasma cells, and occasionally atypical mononuclear cells with mitoses. The epidermal changes are less frequent than those seen in OS, but focal spongiosis and microabscesses may occasionally occur. Moreover, vasculitis with fibrinoid degradation of dermal vessel walls is more frequent in nodular scabies.

Lesions of nodular scabies were previously regarded to be an exaggerated hypersensitivity reaction to the mite and its products, rather than by an active infestation, because mites were rarely found by the mineral oil test or routine skin biopsy (Mittal et al., 1997). However, Suh et al. (2014) suggested a need to differentiate nodular scabies with active infestation from that without active infestation. The authors stated that high-frequency detection of mites in nodular scabies was thought to be associated with the use of dermoscopy, which identified the specific findings of scabies and allowed determination of the proper site for biopsy. Dermoscopic findings such as “delta wing jet” sign (the appearance of a brown triangle that corresponds to the head and the two anterior pairs of legs of the mite) or “jet with contrail” (the appearance of a brown triangle in addition to the white S-shaped burrows which are filled with eggs and scybalas) are considered to be features specific to scabies (Micali et al. 2016).

#### Bullous scabies

It is a rare variety of scabies that usually affect elderly males. Macroscopically, lesions usually mimic those of bullous pemphigoid and manifest as highly irritating bullae that could be flaccid or tense, with or without the classic signs of scabies. Several mechanisms have been proposed to explain bullae formation in scabies. Like bullous impetigo, bullous scabies can result from secondary infection of the scabietic lesions with *Staphylococcus aureus* (Luo et al. 2016). Although bullous scabies may resemble bullous impetigo, presence of mites, eggs, or scybala on direct microscopy or histological sections is diagnostic (Salame and Holland 2019).

In some cases, lesions are associated with anti-basement membrane zone (BMZ) autoantibodies, which are consistent with the diagnosis of bullous pemphigoid. *Sarcoptes scabiei* mites are thought to produce certain enzymes and bullous pemphigoid-like antigens, a phenomenon of antigen mimicry, that induce BMZ antibodies formation, causing complement cascade activation, dermo-epidermal separation and BMZ lytic destruction. The presence of mites within the bullae could support this hypothesis (Kokubu et al. 2019).

#### Crusted scabies

Macroscopically, CS is characterized by formation of yellowish-brown, yellowish-green, or gray crusts that are firmly adherent to the underlying tissues and have a porous appearance when removed. There might be fissuring over the extensor surface of joints. Itching is minimal or even absent (Walton et al. 2010). Crusts are typically seen on the scalp, ear, extensor aspect of elbows, and soles. Nails, like psoriatic nails, exhibit subungual hyperkeratosis and are a major source of relapse (Last et al. 2018). It can occasionally manifest as erythroderma, psoriasis, eczema, seborrheic dermatitis, and pityriasis rubra pilaris (Roberts et al. 2005). However, the diagnosis can be confirmed by microscopic examination of skin scrapings. Davis et al. (2013) developed a grading system for CS based on distribution and extent of crusts, history of previous attacks, and skin condition. The grades were classified as mild (grade 1, score 4–6), moderate (grade 2, score 7–9), or severe (grade 3, score 10–12) (Table 2).

**Table 2:** Grading system for CS (Davis et al. 2013)

A: Distribution and extent of crusting	1	Wrists, web spaces, feet only (< 10% Total Body Surface Area)
	2	As “1” + forearm, lower legs, buttocks, trunk (or 10–30% TBSA)
	3	As “2” + scalp (or > 30% TBSA)
B: Crusting/Shedding	1	Mild crusting (< 5mm depth), minimal skin shedding
	2	Moderate (5-10mm) crusting, moderate skin shedding
	3	Severe (> 10mm), severe skin shedding
C: Past episodes	1	No previous episodes
	2	1–3 hospitalizations for CS (or depigmentation of elbow, knees)
	3	> 4 previous hospitalizations (or depigmentation as above + legs/back, or residual skin thickening)
D: Skin condition	1	No cracking or pyoderma
	2	Multiple pustules or weeping sore or superficial skin cracking
	3	Deep skin cracking with bleeding, widespread exudates
Grading: - Grade 1: total score 4–6 - Grade 2: total score 7–9 - Grade 3: total score 10–12 Treatment: - Grade 1: 3 doses of IVM (days 0, 1, 7) - Grade 2: 5 doses of IVM (days 0, 1, 7, 8, 14) - Grade 3: 7 doses of IVM (days 0, 1, 7, 8, 14, 21, 28)		

**Fig. 3:**
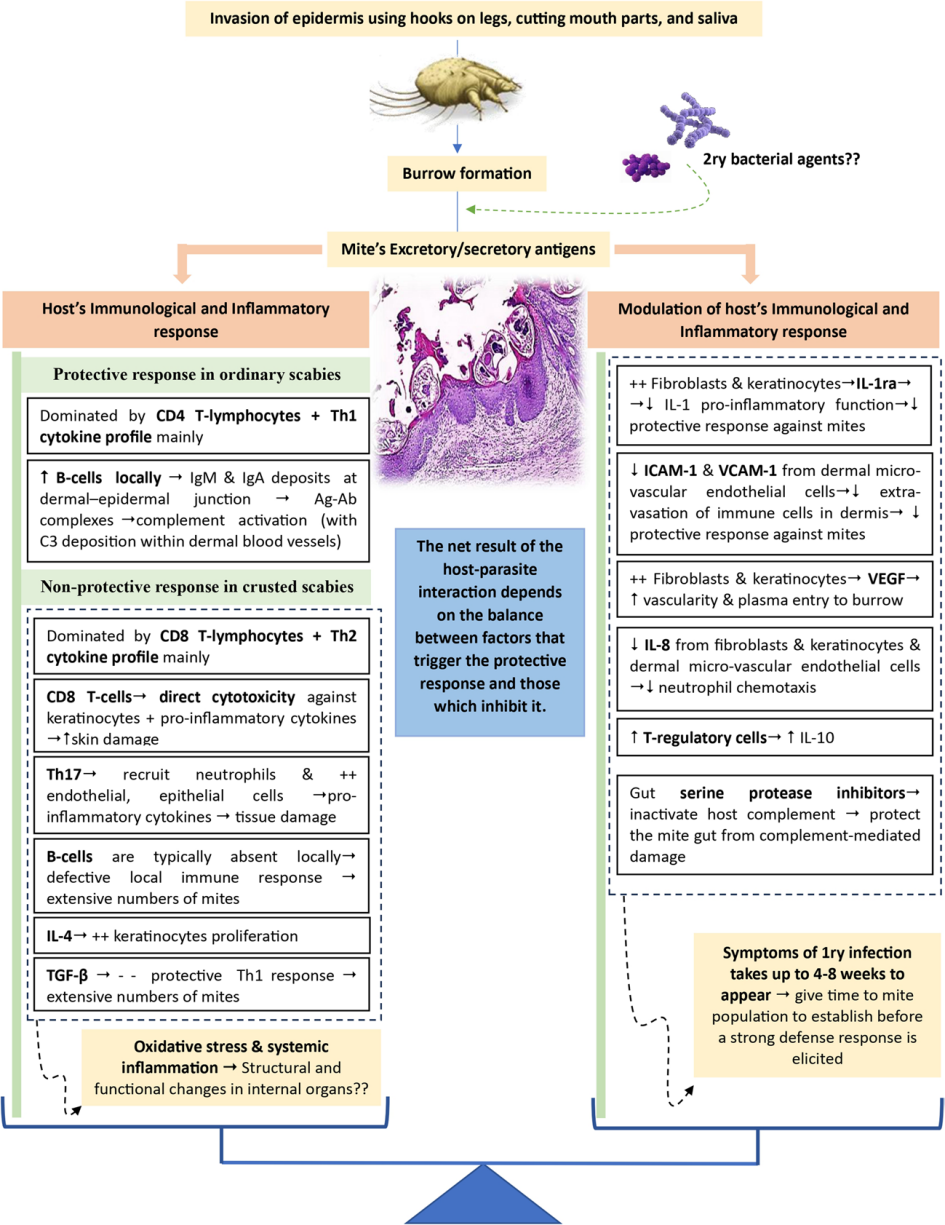
Pathogenesis of scabies

Microscopically, CS is characterized by marked orthokeratotic hyperkeratosis, parakeratosis, irregular acanthosis, and stratum basale hyperplasia. Sebaceous glands and hair follicles are usually hyperplastic, blocked, and inflamed. The epidermis has many burrows in which numerous mites, eggs, larvae, and fecal pellets reside. Also, serum crusts and extravasated erythrocytes are frequent findings. The dermis usually shows superficial and deep perivascular lymphohistiocytic infiltrates with many eosinophils, plasma cells and neutrophils (Sharaf et al. 2023b) (Fig. 3).

#### Infantile scabies

Because of their weak pincer grip and inability to scratch properly, infants with scabies usually have few excoriations, and many burrows. Burrows often show signs of inflammation and are associated with papulovesicular and nodular lesions. Pustules and hemorrhagic crusts may also occur (Hill and Cohen 2017). The mite burden is greater than that in classic scabies in older patients, with several hundred mites found in one patient. In addition, as compared to older children and adults who show with widely disseminated lesions, the early eruption in infants is frequently localized.

Infants have been reported to have more nodules and lower limb involvement, particularly of the soles, than older children and adults with scabies. However, involvement of the scalp and face is also common (Karthikeyan 2007; Boralevi et al. 2014; Eshagh et al. 2014). Inadequate diagnosis might result in superficial skin infections, ecthyma, and cellulitis. Infantile scabies should be distinguished from other similar diseases that may manifest at this age, such as papular urticaria, atopic dermatitis, and infantile acropustulosis (Hill and Cohen 2017).

#### Scabies in elderly

Burrows on the soles of the feet are more common in elderly patients. The finger webs may be completely spared. In contrast to the younger population, scalp and face involvement is common (Berger and Steinhoff 2011). Old patients are more likely to get CS due to various factors in this age group, such as altered immune response, nutritional deficiencies, cognitive disturbances, and inability to maintain personal hygiene (Anderson and Strowd 2017).

#### Scabies incognito

When topical or systemic corticosteroids are used to treat scabies, they obscure the typical itching and inflammation. These cases commonly occur in patients with good hygiene and are referred to as scabies incognito. In such cases, the diagnosis might be readily confused with other skin conditions (Yoshinaga et al. 2009). Although symptoms are masked, the patient remains infectious to others. It is critical to get an accurate diagnosis of scabies incognito since misdiagnosis could lead to dangerous effects such as lesion spreading and superinfection, which can occasionally be fatal (Herwanto et al. 2019).

#### Animal scabies

Occasional cases of scabies have been reported following prolonged human exposure to mangy animals. However, patients usually present with a totally different picture from that which is produced by the *S. scabiei* var *hominis* infection. Because each strain of the mite usually prefers one type of host and does not survive for a long time or reproduce away from it, humans are unlikely to get full-blown scabies from this animal source. In most cases, the infection is self-limiting, with a short incubation period and transient symptoms. As a result, if a patient is suspected to have animal scabies, no treatment of the patient's human contacts is required (Walton and Currie 2007).

Since these regions are exposed to *S. scabiei* mites when the affected animals are carried, lesions are usually found on forearm, lower chest, abdomen, and thighs. Interestingly, both interdigital spaces and genitalia are usually spared. Furthermore, the characteristic deep and long burrows are not formed. Instead, mites burrow into the skin to lay eggs for a short time, inducing itching and severe irritation. Secondary bacterial infection may occur, leading to complications like acute post streptococcal glomerulonephritis (Bandi and Saikumar 2013).

### Systemic changes

**Fig. 4:**
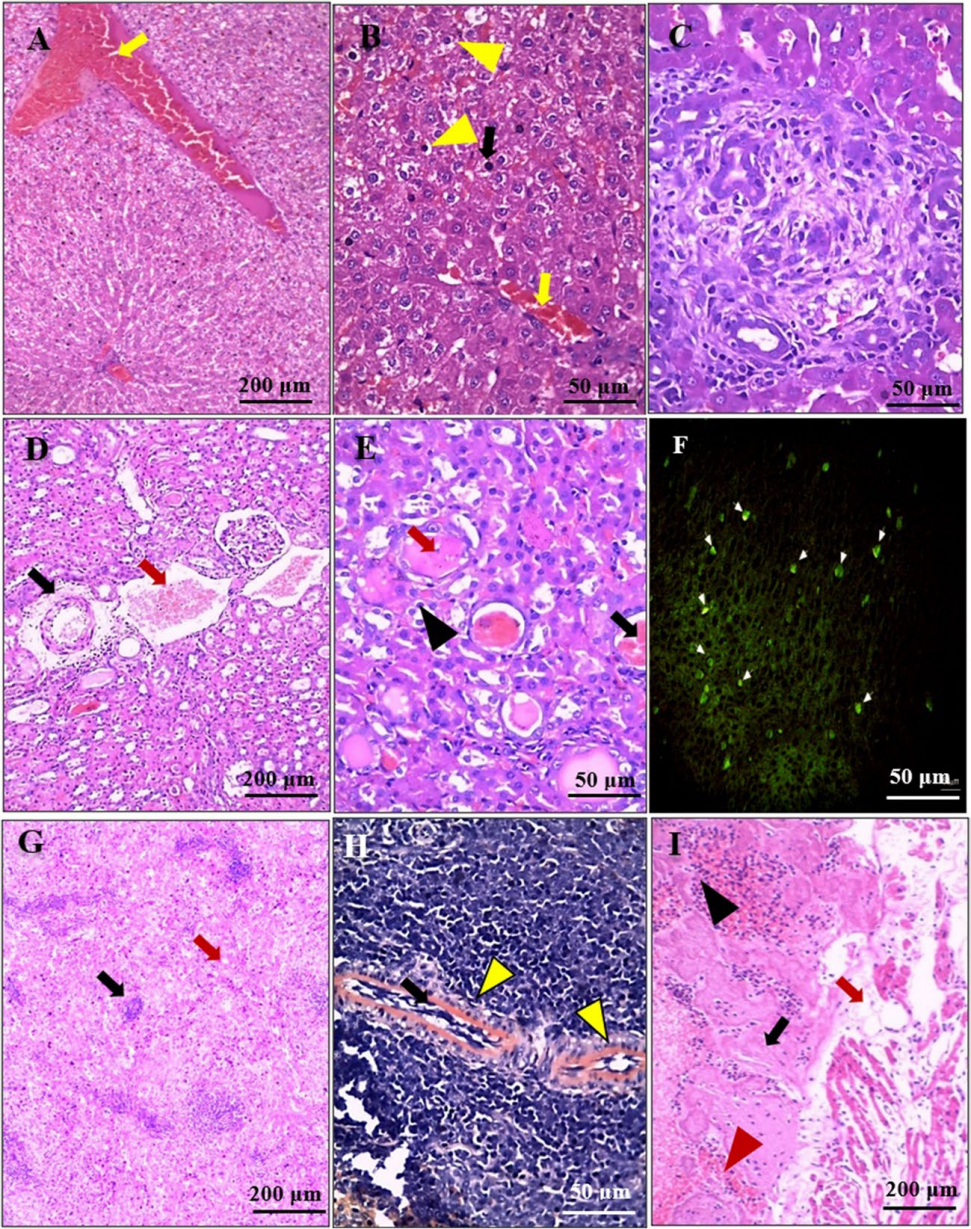
Photomicrographs of tissue sections from internal organs of rabbits with crusted scabies. Figures (**A**, **B**, and **C**) represent liver sections and show marked hepatic congestion "yellow arrows"(**A**, **B**), various degrees of degenerative changes as hydropic degeneration "yellow arrowhead" and pyknotic nuclei of hepatocytes "black arrow" (**B**), and mild to moderate periportal inflammation and fibrosis "red arrows" (C). Figures (**D**, **E**, and **F**) represent renal sections and show severe renal congestion "red arrow", thick-walled renal vessels "black arrow" (**D**); tubular casts "red arrows", vacuolar and hydropic degeneration of tubular cells "black arrowhead"; tubular hemorrhage "black arrow" (**E**); and a bright greenish yellow fluorescence at the sites of immune complex deposition (**F**). Figures (**G** and **H**) represent splenic sections and show moderate to severe splenic congestion in red pulp "red arrow", white pulp atrophy "black arrow" (**G**), and peri-vascular amyloid deposits "yellow arrowheads" (**H**) (H&E: A–E, G, I; IF anti- IgG: F; Congo red: **H**) (Sharaf et al. 2023a)

Despite the availability of wide-ranging data on the local pathological changes in scabies, there are still few studies that address the pathological changes that occur in the non-dermal organs. In a recent study performed on severely affected mangy New Zealand rabbits, Sharaf et al. (2023a) reported definite structural changes observed at the systemic level, evidenced by histopathological changes (severe congestion, different forms of degenerative changes, and inflammatory infiltration) in hepatic, renal, cardiac, and splenic tissue samples (Fig. 4).

In another study performed on severely affected mangy Iberian ibex, authors reported that the most prominent histological findings in the non-dermal tissues were lymphoid hyperplasia, congestion, and presence of amyloid deposits. Additionally, a wide variety of bacterial agents were isolated from lung, liver, spleen, and kidney, indicating a state of septicemia (Espinosa et al. 2017). Interestingly, Valldeperes et al. (2021) reported that no significant changes were observed in sections taken from the heart, kidney, spleen, brain, liver, skeletal muscles, and tonsils obtained from a mangy wild boar. It appears that pathological changes reported in the non-dermal tissues possibly occur only in severe forms of sarcoptic mange (Abdelaziz et al. 2022).

## Complications

The primary scabetic lesions that have been eroded and excoriated by scratching result in reduced skin barrier function and secondary bacterial infection, commonly *Staphylococcus aureus* and group A *Streptococcus* (GAS) (Tasani et al. 2016). Secondary infection with *Staphylococcus aureus* may lead to superficial acute impetiginization, abscesses, cellulitis, echthyma, paronychia, and furunculosis. The most prevalent symptoms of superficial impetiginization are golden purulent/serous crusting or flaccid pustulation. Furthermore, it can also cause osteomyelitis, endocarditis, and potentially fatal bacterial sepsis. Screening for methicillin-resistant *Staphylococcus aureus* (MRSA) and choosing proper medications are crucial for better management of cases (Romani et al. 2015b; Yeoh et al. 2016; Lima et al. 2017; Engelman and Steer 2018).

Secondary bacterial infection caused by GAS can result in acute local skin and soft tissue infections ranging from superficial pyoderma, skin abscesses, and cellulitis to more severe necrotizing fasciitis. Following secondary skin infections, some patients may develop complications such as acute post-streptococcal glomerulonephritis (APSGN) and acute rheumatic fever (ARF), which are associated with chronic renal impairment and chronic rheumatic heart disease, respectively (Carapetis et al. 2005; Steer et al. 2009; Cox et al. 2021). Although GAS pharyngitis was traditionally thought to be the only source of ARF, GAS impetigo has been reported to have a role in ARF (Bowen et al. 2015; Romani et al. 2015a).

According to Sharaf et al. (2023a), scabies could act like a systemic syndrome that may affect other organs beyond the skin. Such syndrome is more likely to occur in severe cases of scabies. According to the authors, it appears that mite’s antigens and the secondary bacterial agents could induce different immune responses and inflammatory reactions, with subsequent alteration of the redox status in the affected host. The resulting oxidative stress condition could induce damage in different tissues, with subsequent changes in systemic serum biochemical and immunological parameters.

Scabies has also psychosocial implications. Since scabies induce severe itching, it can also affect sleep and quality of life of the patient. Scabies places a significant financial burden on low-income families owing to the cost of treatment, school absenteeism, and missed workdays. Hence, limiting their capacity to care for their families and offer educational opportunities for their children (Steer et al. 2009; Engelman et al. 2013; Walker et al. 2017). Furthermore, continuous itching might disrupt a child's sleep, resulting in daytime fatigue, impaired concentration, and decreased productivity (Leung et al. 2020).

Scabies can be stigmatizing for children and their caregivers since it is closely related with poverty and overcrowding and is viewed as a disease of disadvantage and poor hygiene (Jin-gang et al. 2010; Mitjà et al. 2017). Breaking down community misperceptions is still an ongoing issue (Steer et al. 2009; Romani et al. 2015b).

## Conclusions

The host–parasite interaction in scabies is highly complex and involves different mechanisms, some of which are yet largely unknown. Elucidation of the nature of such interaction as well as the underlying mechanisms could allow a better understanding of the mite’s biology and the development of novel diagnostic and therapeutic options for scabies control programs. Moreover, identification of the molecular basis of such interaction could unveil novel targets for acaricidal agents and vaccines.

## Data Availability

Not applicable.

## Abbreviations

*APSGN*: Acute post-streptococcal glomerulonephritis
*ARF*: Acute rheumatic fever
*CS*: Crusted scabies
*GAS*: Group A *Streptococcus*
*IFN-γ*: Interferon gamma
*IL*: Interleukin
*LCs*: Langerhans cells
*MRSA*: Methicillin-resistant *Staphylococcus aureus*
*OS*: Ordinary scabies
*S*: *Sarcoptes*
*TGF-β*: Transforming growth factor beta
*TNF-α*: Tumor necrosis factor alpha.
